# Increased susceptibility of 129SvEvBrd mice to IgE-Mast cell mediated anaphylaxis

**DOI:** 10.1186/1471-2172-12-14

**Published:** 2011-02-03

**Authors:** Muthuvel Arumugam, Richard Ahrens, Heather Osterfeld, Leah C Kottyan, Xun Shang, John A Maclennan, Nives Zimmermann, Yi Zheng, Fred D Finkelman, Simon P Hogan

**Affiliations:** 1 Division of Biochemistry, National Institute of Siddha, Chennai, India; 2 Division of Allergy and Immunology, Department of Pediatrics, University of Cincinnati College of Medicine, Cincinnati Children's Hospital Medical Center, 3333 Burnet Ave, Cincinnati, 45229-3039, USA; 3 Division of Immunobiology, Department of Pediatrics, Cincinnati Children's Hospital Medical Center, 3333 Burnet Ave, Cincinnati, 45229-3039, USA; 4 Division of Experimental Hematology, Department of Pediatrics, Cincinnati Children's Hospital Medical Center, 3333 Burnet Ave, Cincinnati, 45229-3039, USA; 5 Department of Molecular and Cellular Physiology, University of Cincinnati, 231 Albert Sabin Way, Cincinnati, 45267, USA

## Abstract

**Background:**

Experimental analyses have identified strain-dependent factors that regulate susceptibility to anaphylaxis in mice. We assessed the susceptibility of the widely used 129SvEvBrd (also known as 129S5) mouse strain to IgE/mast cell-mediated anaphylaxis as compared to BALB/c. Mice were subjected to passive and oral Ovalbumin [OVA]-induced active anaphylaxis. Tissue mast cell, plasma histamine, total IgE and OVA-specific IgE levels and susceptibility to histamine i.v infusion were assessed. Bone marrow mast cell (BMMC)s were examined for Fc_ε_RI, c-kit, degranulation efficiency, proliferation, apoptosis and cytokine profile.

**Results:**

129S5 mice had significantly increased susceptibility to passive and oral OVA-induced active anaphylaxis. Increased susceptibility to anaphylaxis was associated with increased homeostatic mast cell levels but not OVA-specific IgE or IgG_1 _levels. *In vitro *analyses of BMMCs revealed no difference in Fc_ε_RI and c-Kit expression, however, 129S5 BMMCs possessed greater proliferative capacity and reduced caspase-3-mediated apoptosis. IgE-BMMC degranulation assays demonstrated no difference in degranulation efficiency. Furthermore, 129S5 mice possessed increased sensitivity to histamine-induced hypothermia.

**Conclusions:**

We conclude that 129S5 mice have increased susceptibility to anaphylaxis as compared to BALB/c strain and their increased susceptibility was associated with altered mast cell proliferation and homeostatic tissue levels and responsiveness to histamine. Given the wide spread usage of the 129SvEvBrd strain of mice in experimental gene targeting methodology, these data have important implications for studying IgE-reactions in mouse systems.

## Background

Anaphylaxis is a rapid, life threatening allergic reaction often triggered by food, drugs, insect venoms, latex, or allergen immunotherapy [1-5]. Early clinical and experimental evidence suggests that systemic anaphylaxis is mediated by IgE/mast cell degranulation and the rapid release of preformed mediators, including histamine, proteoglycans, PAF, serotonin, tryptase, chymase and lipid-derived mediators (PGD_2 _and LTC_4, _LTD_4 _and LTE_4_) [6,7]. These mediators are reportedly acting on target cells to induce vasodilation, increased vascular permeability, hypotension, bronchospasm and, as a result, shock.

With the recent emergence of several gene knockout mice (Fc_Є_RI, histamine decarboxylase, IL-4Rα) there has been significant focus on employment of animal models of IgE-mediated passive and active systemic and oral antigen-induced anaphylaxis in an attempt to decipher the relative contributions of inflammatory cells and cytokines to disease pathogenesis [6,8]. These analyses, while identifying important roles for specific molecules in IgE-mast cell mediated reactions, have identified strain-dependent factors that can influence mast cell responses [9]. Moreover, IgE-activation of bone marrow-derived mast cells (BMMCs) derived from C57BL/6 and BALB/c mice promotes the release of different preformed and newly synthesized mediators [10]. Furthermore, differences in susceptibility to food allergy in C3H/HeJ and BALB/c mice were associated with differential Th2 - Th1 responses [11].

In the present study we examined susceptibility of the widely utilized 129SvEvBrd (also known as 129S5) mice to IgE-mediated passive anaphylaxis and oral OVA-induced active anaphylaxis. We demonstrated increased susceptibility to IgE-mast cell-mediated anaphylaxis in the 129S5 mice compared with BALB/c mice. We showed that susceptibility was associated with increased mast cell proliferative capacity, homeostatic tissue mast cell levels and responsiveness to histamine. Given the wide spread usage of embryonic stem cells from the 129S5 strain of mice for generation of various gene knockout mice [12-17] these data have important implications for studying IgE-mast cell reactions in mouse systems.

## Methods

### Animals

6-8 week old weight- and sex-matched 129SvEvBrd mice (Lexicon Genetics, The Woodlands, Texas, USA ) and BALB/c (originally provided by The Jackson Laboratory, Bar Harbor, Maine, USA) mice were maintained in a barrier facility, and animals were handled under IACUC-approved protocols from Cincinnati Children's Hospital Medical Center.

### Passive anaphylaxis model

IgE-mediated anaphylaxis was induced by intravenous (i.v.) injection of rat IgG_2a _anti-mouse IgE mAb (rat IgG2a, clone: EM-95) (10 μg/200 μl saline) and IgG mediated anaphylaxis was induced by intravenous injection of rat IgG_2b _anti-mouse FcγRII/III mAb (rat IgG2_b_, clone: 2.4G2) (500 μg/200 μl saline) as we have previously described [18]. Rectal temperature was monitored for 60 minutes with a rectal probe (Physitemp Model BAT-12) as previously described [18]. Concentration of anti-mouse IgE and anti- mouse FcγRII/III mAb used were determined in preliminary dose-response experiments and also as we have previously demonstrated [19,20].

### Experimental oral antigen-induced active anaphylaxis

6-8 week old mice were sensitized subcutaneously with 50 μg of ovalbumin [OVA] (Sigma-Aldrich, St. Louis, MO) in the presence of 2 mg of aluminum potassium sulfate adjuvant [alum: AIK(SO_4_)_2_-12H_2_O] (Sigma-Aldrich, St.Louis, USA) in sterile saline. Two weeks later, mice were deprived of food for 4 hours and received intragastric (i.g) challenge of ovalbumin [OVA] (50 mg/250 μl saline) employing i.g. feeding needles (Fisher Scientific Co., Pittsburgh, Pennsylvania, USA). Rectal temperatures and diarrhea were monitored at 30 and 60 minutes as we have previously described [18].

### Effect of anti-histamine treatment

To assess the effect of anti-histamine treatment on oral OVA-induced active anaphylaxis, 30 minutes prior to the OVA challenge, mice were i.p injected with H1 and H2 receptor antagonists 0.2 mg of Triprolidine and 0.2 mg of Cimetidine in 200 μl saline and subsequently received OVA via oral gavage, after which rectal temperature was monitored at 30 minutes and 60 minutes time points as we have previously described [18].

### Histamine-induced hypothermia

Histamine biphosphate monohydrate (Sigma-Aldrich, St.Louis, USA) (25 μg/ml saline per mouse) was i.v injected and body temperature was monitored by rectal thermometry every 10 minutes for 60 minutes as we have previously described [18].

### Mast cell quantification

Ear, tongue and jejunum (7 - 10 cm distal to the stomach) were collected and fixed in 10% formalin and processed by standard histological techniques. Longitudinal sections (5 μm) were stained for mast cells with chloroacetate esterase (CAE) activity, as described previously [18]. At least four random sections per mouse were analyzed. Quantification of stained cells was performed by counting the number of chloroacetate-positive cells in 5 fields for tongue, 10 fields for ear and 20 fields for intestine (magnification 400 X).

### BMMC generation

Bone marrow-derived cells (BMMCs) isolated from 6-8 week old mice were maintained in complete medium consisting of RPMI 1640 supplemented with 10% fetal bovine serum (Invitrogen, Carlsbad, USA), glutamine (2 mM), penicillin (100 U/ml), streptomycin (100 mg/ml), pyruvate (1 mM), nonessential amino acids, hepes (10 mM), and 2-ME (50 μM) [21]. BMMCs were cultured in the presence of IL-3 (20 ng/ml) (Peprotech, Inc. Rocky Hill, USA) for the first three weeks, and with both IL-3 (20 ng/ml) and SCF (10 ng/ml) (Peprotech, Inc. Rocky Hill, USA) during week 4. After four weeks of culture, BMMCs were examined for Fc_ε_RI, and c-Kit expressions by flow cytometry analyses as we have previously demonstrated [18,21] and were found to be double positive (95-97%) for Fc_ε_RI and c-Kit at this time point. 4-8 weeks old BMMCs were used for all experiments.

### Peritoneal mast cell analyses

Mice were anesthetized and a hypodermic needle were inserted into the abdominal cavity (18 gauge). Sterile saline (5 ml) was injected through a needle placed near the sternum and the abdominal cavity was gently massaged for 1 minute and the fluid removed. The peritoneal lavage was centrifuged at 1200 rpm for 5 minutes at room temperature. The supernatant was poured off and the cell pellet resuspended in FACS buffer (PBS/1% FCS). The single-cell suspensions were washed with FACS buffer (PBS/1% FCS) and incubated with combinations of the following Abs: PE anti-mouse-FcεRI (clone R35-72; 0.5 μg/10^6 ^cells; BD Pharmingen; San Jose, CA), FITC anti-mouse FcγRII/III (clone 2.4G2; 0.2 μg/10^6 ^cells; BD Pharmingen; San Jose, CA), APC-Alexafluor-750 anti-mouse CD117 (c-kit) (0.2 μg/10^6 ^Ebioscience; San Diego, CA). The following Abs were used as appropriate isotype controls: FITC rat IgG2a and APC-AlexaFluor 750 rat IgG2a (BD Pharmingen; San Jose, CA). Cells were analyzed on FACSCalibur (BD Immunocytometry Systems; San Jose, CA), and analysis was performed using Flow Jo software (Tree Star; Ashland, OR).

### Degranulation Assay

BMMC degranulation was measured by hexosaminidase release as described previously [22]. BMMCs (5 × 10^5 ^cells each) were sensitized with IgE anti-TNP mAb (10 μg/ml) (BD Biosciences, San Jose, CA) for 2 hours. Sensitized BMMCs in 100 μl suspension were challenged with BSA-TNP (10, 100 or 1000 ng/ml) (Biosource Technologies, USA) in different concentrations for 15 minutes. Supernatant was examined for hexosaminidase activity by colormetric determination using 4-Nitrophenyl N-acetyl-β-D-glucosaminide, PNAD (Sigma, St.Louis, USA) at 405 nm. Degranulation is expressed as the percentage of total hexosaminidase activity. The BMMC lysate was used to estimate total hexosaminidase activity. In some experiments BMMCs (5 × 10^6^/ml) were sensitized with IgE anti-TNP mAb (10 μg/ml) for an hour, and challenged with BSA-TNP (100 ng/ml) for 3 hours. Supernatant was analyzed for IL-6, IL-13 and TNF-α by ELISA as described by manufacturer (R&D systems, Minneapolis, USA).

### Proliferation Assay

BMMCs (2 × 10^6^) were stained with CFSE (5 μM) (Invitrogen, Carlsbad, USA) for 10 min in PBS with 0.1% BSA and cultured in the presence of IL-3 (20 ng/ml) and SCF (10 ng/ml). The cells were examined by flow cytometry for CFSE mean fluorescence intensity on the 4^th ^and 8^th ^day. CFSE positive cells were analyzed with a proliferation software tool (Flowjo, Ashland, USA) [23].

### Apoptosis

Apoptosis was determined using annexin V-allophycocyanin and 7-aminoactinomycin D (7AAD), as we have previously described [24]. Early apoptosis was defined as annexin V-positive, 7AAD negative. Briefly, BMMCs (2 × 10^6^) were washed and resuspended in annexin V-binding buffer and stained with annexin V-allophycocyanin and 7AAD. Samples were then incubated at room temperature for 15 minutes and analyzed by flow cytoemetry. Active Caspase 3 expression, a biomarker for cells in apoptosis, was examined by flow cytometry using PE-Rabbit Anti-Active Caspase 3 antibody (BD Pharmingen, San Diego, USA [24].

### ELISA measurements

ELISA measurements for OVA-specific IgG_1 _and IgE were done as previously reported [18]. Total IgE was estimated as described by the manufacturer using a mouse IgE Elisa Kit BD-Opt EIA™ (BD Biosciences, New Jersey, USA). Total IgG_1 _and IgG_2a _concentrations (1/10000) were compared between BALB/c and 129S5 mice. Serum IgG was captured with goat anti-mouse IgG (1:1000 Dilution) (Southern Biotech, Birmingham, USA). After capture, serum IgG_1 _and IgG_2a _were detected by using different secondary antibodies; namely goat anti-mouse IgG_1_-HRP (Southern Biotech, Birmingham, USA) and goat anti-mouse IgG_2a_-HRP (Southern Biotech, Birmingham, USA), respectively.

### Plasma Histamine determinations

Plasma was collected 5 min after anti-IgE-induced passive anaphylaxis (10 μg/200 μl saline i.v). Plasma was collected in EDTA-coated tubes and plasma histamine levels were determined with an histamine immunoassay kit (Immunotech, Marseille Cedex, France) as described by the manufacturer.

### Statistical Analysis

Data are expressed as mean ± standard error of the mean (SEM), unless otherwise stated. Statistical significance comparing different sets of mice was determined by Student's t-test. In experiments comparing multiple experimental groups, statistical differences between groups were analyzed using the one-way ANOVA parametric and a Tukey's multiple comparison post-test. In experiments comparing two experimental groups, statistical differences between groups were determined using a Students T-test. P < 0.05 was considered significant. All analyses were performed using Prism 5.0 software.

## Results

### Increased susceptibility of 129S5 mice to IgE/mast cell-mediated anaphylaxis

**Figure 1:**
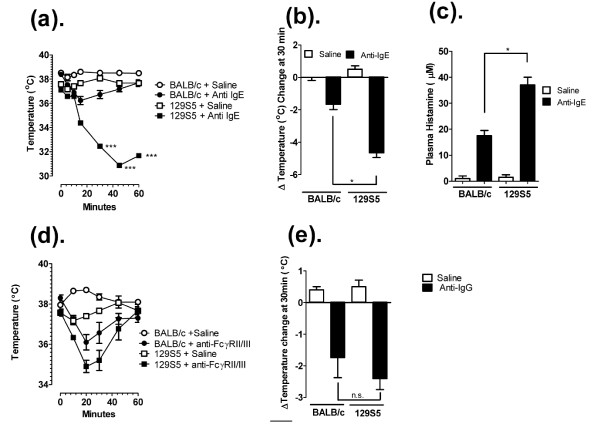
**IgE and IgG passive anaphylaxis in 129S5 and BALB/c mice**. a) Rectal temperature 0-60 minutes and (b) maximal temperature change at 30 minutes and (c) Plasma histamine concentration at 5 minutes in control Ig and anti-IgE-treated (10 μg/200 μl saline) 129S5- and BALB/c mice. d) Rectal temperature 0-60 minutes and (e) maximal temperature change at 30 minutes in control Ig and anti-FcγRII/III-treated (500 μg/200 μl saline) 129S5 and BALB/c mice. (n = 6) Data represent mean ± SEM. Data were analyzed by one-way analysis of variance (ANOVA) and a post-hoc comparison test (Tukey-Kramer). (a) Triple asterisks indicate a P value < 0.001 compared with BALB/c + Anti-IgE. (b, c and e) Single asterisk indicates a P value < 0.05. n.s. not significant.

**Figure 2:**
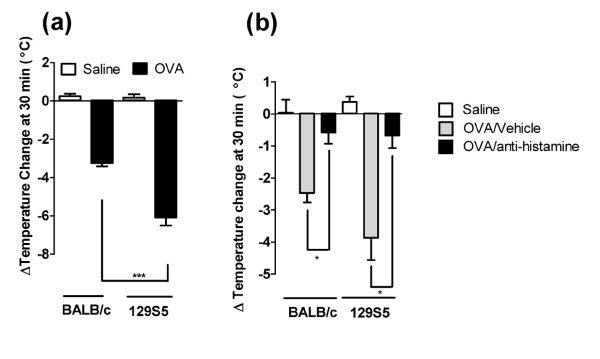
**Oral antigen-induced anaphylaxis in 129S5 and BALB/c mice is histamine-dependent**. (a) Maximal rectal temperature change at 30 minutes following OVA gavage of OVA-sensitized 129S5 and BALB/c mice. (b) Maximal rectal temperature change at 30 minutes following OVA gavage of OVA-sensitized 129S5 and BALB/c mice treated with H1 and H2-receptor antagonists. Mice were sensitized with OVA/Alum (50 μg/2 mg) subcutaneously. Two weeks later, after 4 hours starvation, mice received oral gavage of OVA (50 mg/250 μl) saline and rectal temperatures were recorded at 30 and 60 minutes using rectal thermometry. b) Mice were i.p injected with anti-histamine (0.2 mg Triprolidine + 0.2 mg Cimetidine/200 μl saline) 30 minutes before prior to OVA- challenge and rectal temperatures determined at 30 min and 60 minutes following OVA gavage. (n = 8) Data represent mean ± SEM. Data were analyzed by one-way analysis of variance (ANOVA) and a post-hoc comparison test (Tukey-Kramer). Single and triple asterisks indicate a P value less than < 0.01 and < 0.001, respectively.

**Figure 3:**
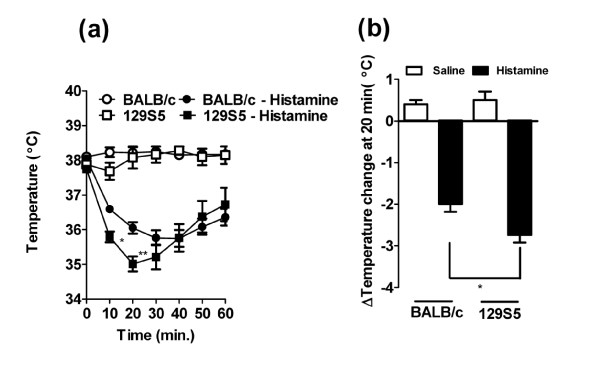
**Histamine-induces enhanced hypothermia in 129S5 mice**. a) Rectal temperature 0-60 minutes and (b). Maximal rectal temperature change at 30 minutes following i.v. injection of histamine (5 mg/200 μl saline). Data presented as means ± SEM (n = 6-8) and is representative of two separate experiments. Data were analyzed by one-way analysis of variance (ANOVA) and a post-hoc comparison test (Tukey-Kramer). (a) Single and double asterisks indicate a P value < 0.05 and < 0.01 compared with BALB/c + histamine. (b) Single asterisk indicate a P value less than < 0.05.

Recently we and others have performed experimental analyses employing murine models of anaphylaxis and mice deficient in specific genes implicated in the regulation of IgE and mast cell function to identify the relative contribution of these molecules to the manifestations of IgE/mast cell-mediated anaphylaxis [15,19,25-32]. These analyses often involve usage of mice of different or mixed strains, in particularly 129S5, a mouse strain frequently used in gene targeting analyses, and the BALB/c, a "Th2-skewed" strain that develop robust allergic phenotypes. We were interested in assessing for the presence of strain-dependent anaphylactic phenotypes between these commonly utilized mouse strains to eliminate possible contribution of strain-dependent factors in studies employing gene targeted mice. To assess this, we initially examined passive IgE-mediated anaphylaxis in 129S5 and BALB/c mice. Intravenous (i.v.) infusion of anti-mouse IgE (EM95) resulted in anaphylaxis as evidenced by hypothermia (Figure 1). Notably, hypothermia was significantly more severe in the 129S5 mice compared to BALB/c (Figure 1a and 1b). To ascertain whether the increased hypothermia was associated with increased mast cell degranulation, we examined plasma histamine levels. Notably, we found significantly elevated plasma histamine levels in 129S5 mice compared to BALB/c (Figure 1c). Importantly, 129S5 mice were not significantly more susceptible to IgG mediated anaphylaxis (Figure 1d and 1e) indicating that this phenotype is specific to IgE/mast cell-mediated reactions. We have previously demonstrated that oral antigen triggered anaphylaxis is mast cell and IgE dependent [18,27]. To assess whether 129S5 mice had increased susceptibility to oral antigen triggered anaphylaxis, OVA-primed 129S5 and BALB/c mice were orally gavaged with OVA and body temperature measured; notably, hypothermia in the 129S5 mice was more severe than that observed in BALB/c mice (Figure 2a). To determine whether oral OVA-triggered hypothermia was histamine-dependent, mice received histamine receptor type-1 and -2 receptor antagonists (Triprolidine and Cimetidine) 30 minutes prior to the OVA challenge. H1 and H2 receptor antagonism blocked the OVA-induced hypothermia in both 129S5 and in BALB/c mice indicating that anaphylaxis was mast cell/histamine dependent (Figure 2b). To assess whether the increased susceptibility to IgE-reactions in 129S5 mice was due to increased sensitivity to mast cell mediators, mice were i.v. injected with histamine and hypothermia was assessed. Injection (i.v) of histamine induced a significant temperature decrease in both 129S5 mice and BALB/c mice, however the decrease in 129S5 mice was significantly greater compared to that in BALB/c mice (Figure 3a and 3b). These studies indicated that 129S5 mice have increased susceptibility to IgE/mast cell/histamine-mediated hypothermia relative to BALB/c mice.

### Serum Ig and mast cell levels in S129S5 mice

**Figure 4:**
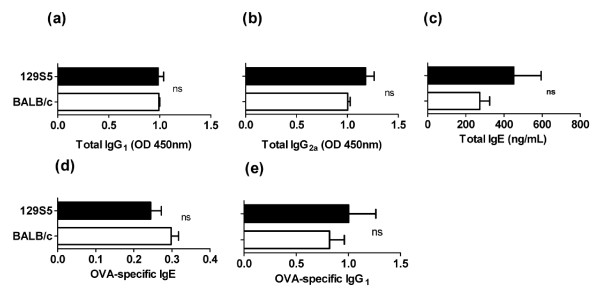
**Antigen-specific and total IgG and IgE levels in 129S5 and BALB/c mice**. Total (a) IgG1, (b) IgG2a and (c) IgE in the serum of naive BALB/c and 129S5 mice. (d) OVA-specific IgE (d) and IgG1 (e) in OVA-sensitized mice. Data represents mean ± SEM. ( n = 4-6) and is representative of two separate experiments. n.s. not significant.

**Figure 5:**
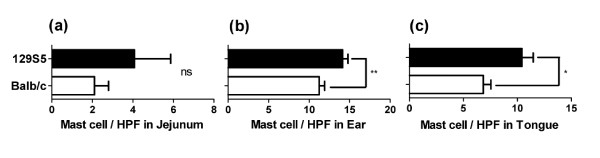
**Increased systemic mast cells in 129S5 mice**. Mast cells/hpf in: a) jejunum, (b) ear skin and (c) tongue of 129S5 and BALB/c mice. Data represent mean ± SEM (n = 4-6) and is representative of two separate experiments. Data were analyzed using a Students T-test. Single and double asterisks indicate a P value < 0.05 and < 0.01 respectively. n.s. not significant.

To determine whether the increased susceptibility was associated with differences in total or antigen specific IgE levels, we assessed serum immunoglobulin levels. The total IgE, IgG_1 _and IgG_2a _levels were comparable between naïve 129S5 and BALB/c mice (Figure 4a-c). Furthermore, i.p. OVA/alum sensitization induced a comparable OVA-specific serum IgE and IgG_1 _responses (Figure 4d and 4e). We next assessed homeostatic tissue mast cell levels, and demonstrated significantly elevated mast cell levels in the ear and tongue, but not the intestine, of the 129S5 mice compared to BALB/c mice (Figure 5a-c). The data indicate that the increased susceptibility is associated with increased tissue mast cell levels.

### BMMC in 129S5 mice

**Figure 6:**
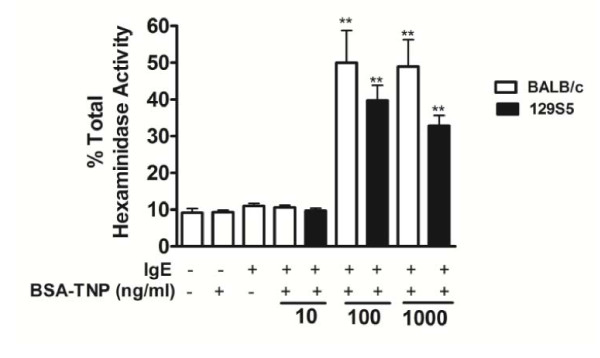
**IgE-mediated BMMC degranulation in 129S5 and BALB/c mice**. (a) β-hexosaminidase activity at 15 minutes following IgE-mediated degranulation of 129S5 and BALB/c BMMC. 4 week-cultured 129S5 and BALB/c BMMCs (5 × 10^6^/ml) were sensitized with IgE-TNP (10 μg/ml) and challenged with BSA-TNP for 15 minutes and supernatant was assayed for hexosaminidase activity as described in materials and methods. Data represents mean ± SEM and is representative of three separate experiments. Data were analyzed by one-way analysis of variance (ANOVA) and a post-hoc comparison test (Tukey-Kramer). Double asterisks indicate a P value < 0.01 compared with negative control (IgE and BSA-TNP negative).

**Figure 7:**
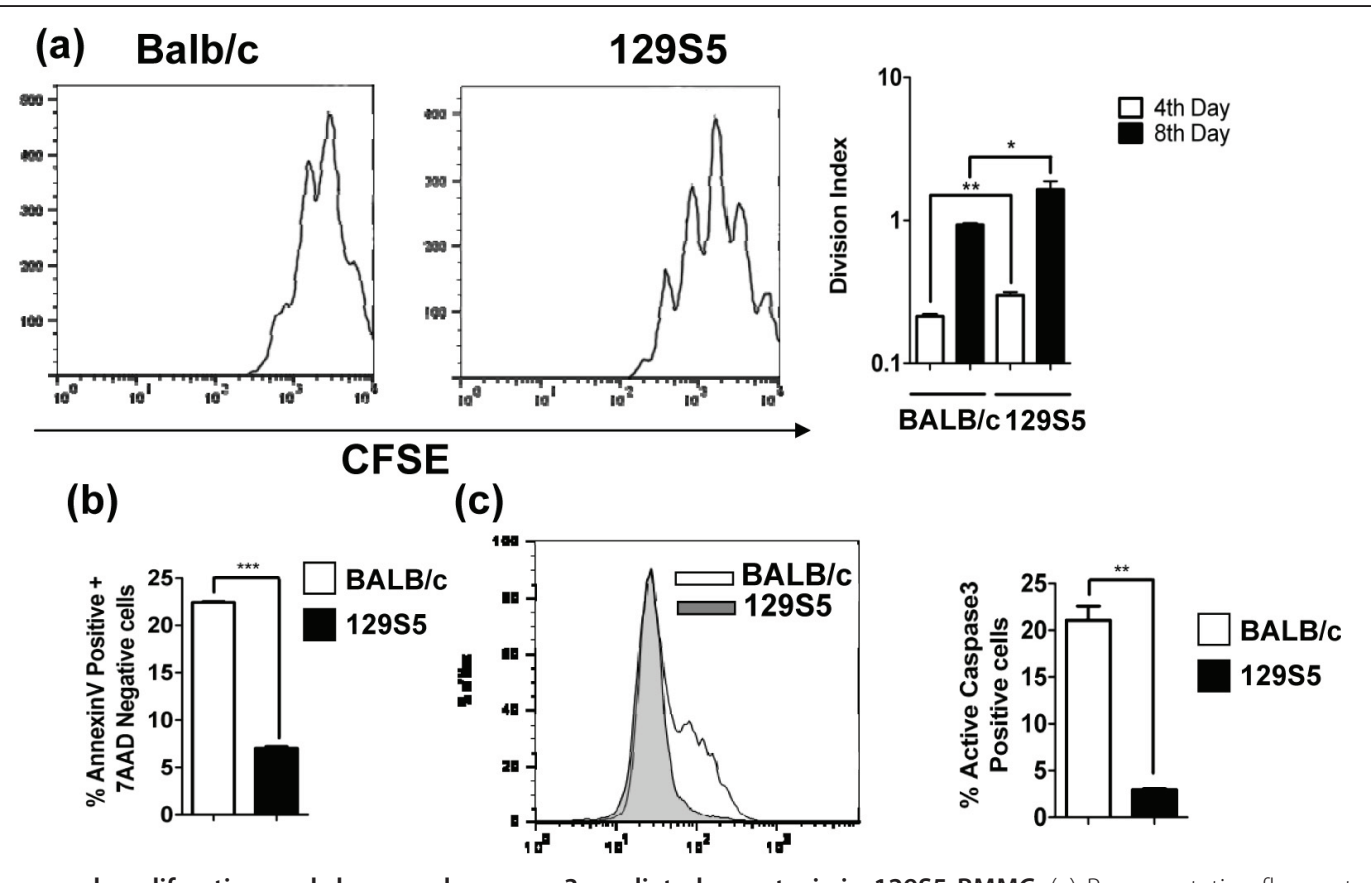
**Increased proliferation and decreased caspase-3-mediated apoptosis in 129S5 BMMC**. (a) Representative flow cytometry plot (day 8) of CFSE-labeled BALB/c and 129S5 BMMCs. Divisional index of 129S5 and BALB/c BMMCs cultured in IL3 (20ng/ml) and SCF (10ng/ml) and CFSE-fluoresence decay was measured on day 4 and day 8 and analyzed by proliferation tool of Flowjo software to determine division index. (b) Percentage of 7AAD- Annexin V^+ ^and (c) active caspase-3^+ ^129S5 and BALB/c BMMCs (5-week old) cultured in IL-3 (20 ng/ml) and SCF (10 ng/ml). Data represent mean ± SEM and is representative of three separate experiments. (a) Data were analyzed by one-way analysis of variance (ANOVA) and a post-hoc comparison test (Tukey-Kramer). Single and double asterisks indicate a P value < 0.05 and < 0.01, respectively. (b and c) data were analyzed using a Students T-test. Double and triple asterisks indicate a P value < 0.01 and < 0.001, respectively.

To define if the divergence in susceptibility to anaphylaxis in 129S5 and BALB/c mice was due to inherent differences in mast cell function, we assessed phenotypic and degranulation properties of BMMCs. Surface expression of Fc_Є_RI and c-Kit on BMMCs from 129S5 and BALB/c mice were comparable (results not shown). Similarly, the level of IgE-mediated degranulation of BMMCs, as measured by β-hexosaminidase activity and cytokine (IL-6, IL-13 and TNFα) release, was also not significantly different between strains (Figure 6a and results not shown). Since we observed increased systemic mast cell levels in 129S5 compared to BALB/c mice, we examined BMMC proliferation rate and apoptosis. Proliferation by 129S5 BMMCs in the presence of IL-3 and SCF was significantly increased relative to BALB/c BMMC (Figure 7a). Furthermore, the level of 129S5 BMMC apoptosis was reduced compared to BALB/c. Specifically, the percentage of BMMCs entering early apoptosis (Annexin V^+ ^7AAD^-^) was significantly reduced in 129S5 BMMCs compared to BALB/c (Figure.7b). Consistent with this observation, active Caspase-3 expression was significantly decreased in 129S5 BMMCs when compared to BALB/c BMMCs (Figure 7c).

## Discussion

129S5 mice demonstrated increased susceptibility to both passive and active IgE-mediated anaphylaxis. The increased susceptibility was linked with elevated homeostatic tissue mast cells and increased sensitivity to histamine infusion. *In vitro *analysis demonstrated 129S5-derived BMMCs have increased proliferative capacity and decreased apoptosis. Collectively, these studies demonstrate that 129S5 mice have inherent differences in bone marrow-derived mast cell functional capacity and sensitivity to mast cell-derived mediators, and that these factors are sufficient to alter susceptibility to IgE mediated anaphylactic reactions.

Systemic and oral antigen-induced IgE-mediated anaphylaxis in mice is dependent on the IgE-FcεRI-mast cell pathway. Ag-IgE crosslinking of the FcεRI on mast cells leads to degranulation and release of preformed mediators, such as histamine, leading to anaphylactic shock [6,8,20,26,33]. Indeed we demonstrated that antagonism of histamine activity blocked the anaphylactic response. Notably, 129S5 mice have increased susceptibility to IgE-mediated anaphylaxis that is linked with elevated systemic mast cells levels. We suggest that activation of a greater number of mast cells leads to increased systemic levels of mast cell mediators, such as histamine, and increase severity of the anaphylactic reaction. Consistent with this, we demonstrated increased serum histamine in 129S5 mice compared to BALB/c. Elevated serum histamine can also be attributed to increased degranulation efficiency of mast cells, but we found no difference in degranulation efficiency (i.e. equivalent hexosaminidase and cytokine release) of BMMCs from 129S5 and BALB/c mice suggesting that mast cell load may explain the observed differences between strains. Consistent with this, previous experimental studies employing IL-9 transgenic mice have demonstrated that elevated systemic mast cell levels increased IgE-induced mast cell mediator release and severity of anaphylaxis [34].

Analyses of BMMC from 129S5 and BALB/c mice revealed differences in mast cell function. Moreover, 129S5 BMMC possessed increased proliferative capacity and reduced apoptosis. The increased proliferative capacity and survival of 129S5 BMMCs could explain the increased systemic mast cell levels in 129S5 mice compared with BALB/c.

We demonstrated that IgE-mediated anaphylaxis is histamine-dependent. Furthermore, we showed increased plasma histamine in 129S5 mice compared with BALB/c mice. Histamine regulates body temperature by acting on hypothalamic histaminergic neurons in the central nervous system [29] or alternatively, and more relevant to a rapid anaphylactic reaction, via induction of vascular leak through dysregulation of endothelial gap formation of the vascular wall [35,36]. Interestingly, 129S5 mice displayed increased susceptibility to intravenous histamine-induced hypothermia, suggesting a strain-dependent difference in vascular permeability. This is consistent with the previously reported strain-dependent differences in pulmonary vascular permeability in response to ischemia [37]. Collectively, our data indicate that strain-dependent differences in tissue mast cell levels attributed to possible increased proliferation and apoptosis rates and in part an increase in histamine sensitivity heighten 129S5 mice to IgE-mast cell-mediated reactions.

Previous investigations have demonstrated that expression levels of the FcγRII can influence IgE-mast cell regulated anaphylaxis [38,39]. Moreover, mice deficient in the FcγRIIB display enhanced IgE-mediated anaphylactic responses [38]. Notably, the 129/Sv and 129/Ola strains of mice share a promoter haplotype associated with reduced expression and function of the FcγRII [40]. To determine if the observed differences in severity of anaphylaxis between 129S5 and BALB/c is due to altered FcγR expression, we assessed FcγRII/III expression on peritoneal mast cells [39]. We observed no significant difference in expression of FcγRII/III on 129S5 peritoneal mast cells (c-kit^+ ^FceRI^+^) compared to BALB/c (FcγRII/III mean fluoresence intensity [MFI] 61.9 ± 9.2 vs 74.4 ± 14.1; mean ±SD; n = 4 per group; not significant) suggesting that the level of FcγRII/III on mast cells is not the primary reason for the observed differences in IgE-mast cell-triggered reactions. Importantly, Takai et al., [31] demonstrated that loss of FcγRII expression was associated with increased IgE-and IgG-triggered anaphylaxis. We did not observe any significant differences in the severity of IgG-mediated anaphylaxis between BALB/c and 129S5 mice further supporting the notion that increased severity of anaphylaxis in 129S5 mice is independent of FcγRII expression.

Previous experimental data has identified strain-dependent differences in mast cell function. Lyn^-/- ^mice on the C57BL6 and BALB/c background demonstrated opposite phenotypes with respect to mast cell degranulation efficiency [9]. Furthermore, BALB/c BMMC-derived PGD synthase, IL-6 and MCP-1 levels are significantly elevated in comparison to C57BL6 [10]. We are unable to assess differences in vivo anaphylactic phenotypes between 129S5, BALB/c and C57BL/6, as C57BL/6 mice are resistant to oral antigen-induced anaphylaxis induced by OVA sensitization and repeated oral challenge (results not shown). The fact that C57BL/6 mice (H-2b haplotype) are resistant to oral antigen-induced anaphylaxis, and the 129S5 (H-2b haplotype) have increased responsiveness compared to BALB/c (H-2d haplotype) indicates that severity of anaphylaxis in these strains is independent of MHC haplotypes. Furthermore, there are approximately 17 sub-strains in the 129 strain, which possess quite different genetic and phenotypic characteristics [41-43]. Thus, it is possible that there are also differences in anaphylaxis phenotypes between 129 substrains that are yet to be identified. Our identification of substantial strain-dependent differences between the mast cells of 129S5 and BALB/c mice has important implications for the study of anaphylaxis and mast cell biology given that embryonic stem cells from 129S5 mice are widely used in experimental gene targeting methodology [42-45]. The strain dependent differences indicate that rigorously designed breeding strategies for control of genetic background must be employed in such gene targeting studies to reduce the influence of strain dependent traits on IgE-mast cell responses in the knock out models.

## Conclusions

In conclusion, higher systemic mast cell load and sensitivity to histamine increases the susceptibility of 129S5 mouse strain to IgE-mast cell mediated anaphylaxis.

## Authors' contributions

MA designed and performed experiments, analyzed and interpreted data and drafted manuscript. RA HO LCK XS NZ performed laboratory experiments. YZ JAM and FF provided reagents, discussed experimental design and interpreted data. SPH designed and performed experiments, analyzed interpreted data and drafted manuscript. All authors read and approved the final manuscript.
